# Up‐regulated acylglycerol kinase (AGK) expression associates with gastric cancer progression through the formation of a novel YAP1‐AGK–positive loop

**DOI:** 10.1111/jcmm.15613

**Published:** 2020-08-22

**Authors:** Shanshan Huang, Yuan Cao, Hui Guo, Yangyang Yao, Li Li, Jun Chen, Junhe Li, Xiaojun Xiang, Jun Deng, Jianping Xiong

**Affiliations:** ^1^ Department of Oncology The First Affiliated Hospital of Nanchang University Nanchang China

**Keywords:** AGK, biomarker, gastric cancer, Hippo‐YAP1 pathway

## Abstract

Acylglycerol kinase (AGK) uses adenosine triphosphate (ATP) and acylglycerol to generate adenosine diphosphate (ADP) and acyl‐sn‐glycerol 3‐phosphate in cells. Recent evidence has demonstrated that dysregulated AGK expression is associated with the development of various human cancers. This study investigated the effects of AGK on gastric cancer cell proliferation and carcinogenesis and explored the underlying molecular events. AGK expression was up‐regulated in gastric cancer and was associated with poor prognosis in gastric cancer patients. AGK overexpression increased gastric cancer proliferation, invasion capacity and the expression of the epithelial‐mesenchymal transition markers in vitro. Conversely, the knockdown of AGK expression reduced gastric cancer cell proliferation in vitro and in nude mouse tumour cell xenografts. Importantly, AGK expression was associated with the YAP1 expression in gastric cancer cells and tissues. YAP1 expression also transcriptionally induced AGK expression through the binding of TEAD to the AGK gene promoter. However, AGK expression inhibited the activation of the Hippo pathway proteins and induced YAP1 nuclear localization to enhance the transcription activity of YAP1/TEADs. In conclusion, the study demonstrates that AGK is not only a novel target of the Hippo‐YAP1 pathway, but that it also positively regulates YAP1 expression, thus forming a YAP1‐AGK–positive feedback loop.

## 1 INTRODUCTION

Gastric cancer is the fifth most prevalent type of cancer and the third leading cause of cancer‐related deaths worldwide.^1^ During gastric tumorigenesis and cancer progression, multiple proteins and gene signalling pathways are altered.^2^, ^3^ Thus, further research investigating the underlying molecular mechanism of gastric tumorigenesis and cancer progression could facilitate the identification of novel prognostic markers and therapeutic strategies to control this deadly disease. Previous studies have demonstrated that the Hippo pathway plays a critical role in the regulation of organ size during embryo development and tissue homeostasis in adults. On the molecular level, for example, when the Hippo pathway is activated, macrophage‐stimulating protein 1/2 (MST1/2) will phosphorylate large tumour suppressor kinase 1/2 (LATS1/2) and the latter will subsequently phosphorylate and repress the activity of Yes‐associated protein 1 (YAP1) and its homolog transcriptional co‐activator TAZ (also known as WWTR1).^4^, ^5^, ^6^ Thereafter, YAP1 that is phosphorylated at S127 or TAZ that is phosphorylated at S89 will bind to the 14‐3‐3 protein, leading to YAP1 and TAZ retention in the cell cytoplasm; otherwise, YAP1 and TAZ proteins will translocate into the nucleus where they activate transcription and the expression of their downstream genes (like CTGF, CYR‐61, FOXM1 and CDX2) to facilitate their biological functions in cells, such as cell proliferation and migration.^7^, ^8^, ^9^ Accumulating evidence suggests that dysregulation of the Hippo pathway signalling is associated with the development, progression and metastasis of different human cancers.^10^, ^11^ Thus, alteration of YAP1 expression and activity has also been shown to be associated with cancer development.^12^, ^13^ In gastric cancer, YAP1 expression contributes to poor patient survival.^14^ YAP1 acts as an oncogene or possesses an oncogenic effect in gastric cancer^15^ and is able to promote gastric cancer cell survival and migration.^16^ Although a few regulatory factors that can act on Hippo‐YAP1 pathway have been uncovered, the mechanism of Hippo inactivation and YAP1 relevant transcriptional targets in GC are still remained incompletely understood.

Acylglycerol kinase (AGK), acting as a lipid kinase, functions to phosphorylate monoacylglycerol and diacylglycerol to form lysophosphatidic acid (LPA) and phosphatidic acid (PA),^17^ resulting in the activation of the downstream signalling.^17^, ^18^, ^19^ Recently, AGK was shown to be a cancer‐related protein that is overexpressed in various human cancers, such as prostate cancer,^20^ hepatocellular carcinoma,^21^ breast cancer^22^ and oesophageal squamous cell carcinoma (ESCC).^23^ The level of AGK expression is significantly associated with the Gleason scores and capsular invasion of prostate cancer,^20^ the angiogenesis and tumour cell survival of hepatocellular cancer,^21^ and the sustained constitutive JAK2/STAT3 activation in oesophageal squamous cell carcinoma.^23^ In human carcinogenesis, both the Hippo‐YAP1 and AGK pathways co‐ordinately play a role, although the precise protein‐protein interactions and molecular pathways require further investigation.

Therefore, in this study, we investigated the effects of AGK on gastric cancer cell proliferation and carcinogenesis and the underlying molecular events. We expected to provide novel information regarding the role of the AGK and Hippo‐YAP1 pathways in the development of gastric cancer and determine whether they should be further evaluated as biomarkers for the early detection and prediction of prognosis in gastric cancer and explored as therapeutic targets for the treatment of gastric cancer.

## 2 MATERIALS AND METHODS

### 2.1 Tissue samples, cell lines and culture

In this study, we collected 120 gastric cancer tissue samples in the form of paraffin blocks from the Department of Pathology, The First Affiliated Hospital to Nanchang University (Nanchang, China). The samples were taken from patients that were hospitalized between January 2009 and December 2012 and were histologically diagnosed with gastric cancer. None of the included patients received any pre‐surgery chemotherapy. The clinicopathological data from each patient were collected from their medical history (Table S1). Tumour stages were classified according to the 2010 criteria of the American Joint Committee on Cancer.^24^ In addition, we obtained 12 fresh gastric cancer and paired non‐cancerous mucosal tissues from the surgery room, which were immediately snap‐frozen in liquid nitrogen and stored at −80°C until use. This study was approved by the Ethics Committee of The First Affiliated Hospital of Nanchang University, and all tissue specimens were collected with the patients' consent.

Human gastric cancer cell lines, SGC‐7901, BGC‐823, MGC‐803, MKN‐45 and HGC‐27, and an immortalized human gastric epithelial cell line, GES‐1, as well as a HEK‐293T cell line were obtained from the Shanghai Institute of Life Science, Chinese Academy of Sciences (Shanghai, China). These cell lines were cultured in Dulbecco's modified Eagle's medium (DMEM; Gibco, Grand Island, NY) supplemented with 10% foetal bovine serum (FBS; Thermo Fisher Scientific, Waltham, MA, USA) at 37°C in a humidified incubator with 5% CO_2_.

### 2.2 Protein extraction and Western blot

Protein was extracted using a radioimmunoprecipitation assay (RIPA) buffer and quantified. Protein samples, with 15‐30 µg per loading, were separated in sodium dodecyl sulphate‐polyacrylamide gel electrophoresis (SDS‐PAGE) gels and transferred on to polyvinylidene fluoride membranes (PVDF; Millipore, Billerica, MA, USA). For Western blotting, we used the following primary antibodies; rabbit polyclonal anti‐AGK (GTX107413, GeneTex, Irvine, CA, USA, 1:1000); rabbit monoclonal anti‐YAP (D8H1X; #14074, Cell Signaling Technology, Danvers, MA, USA, 1:1000); rabbit monoclonal anti‐MST1 (D8B9Q; #14946, Cell Signaling Technology, 1:1000); rabbit polyclonal anti‐MST2 (#3952, Cell Signaling, 1:1000); rabbit monoclonal anti‐LATS1 (C66B5; #3477, Cell Signaling Technology, 1:1000); rabbit monoclonal anti‐LATS2 (D83D6; (#5888, Cell Signaling Technology, 1:1000); rabbit polyclonal anti‐phospho‐YAP (Ser127; #4911, Cell Signaling Technology, 1:1000); rabbit polyclonal anti‐CTGF (GTX124232; GeneTex, 1:1000); rabbit monoclonal anti‐vimentin (D21H3; #5741, Cell Signaling Technology, 1:1000); rabbit polyclonal anti‐fibronectin (ab2413; Abcam, Cambridge, MA, USA, 1:1000); rabbit polyclonal anti‐N Cadherin (ab18203; Abcam, 1:1000); mouse monoclonal anti‐E Cadherin (ab1416; Abcam, 1:1000); and mouse monoclonal anti‐GAPDH (GTX627408; GeneTex, 1:1000). Antibodies were used according to the manufacturer's protocols.

### 2.3 Immunohistochemistry

Immunostaining of YAP1 and AGK proteins in paraffin sections of the gastric cancer tissues followed a protocol that was described in a previous study.^25^ The staining results were evaluated by two pathologists, based on the proportion of positively stained cells and the intensity of staining, that is % of staining was scored as 0 (0%), 1 (0%‐10%), 2 (10%‐50%) and 3 (50%‐100%), while the intensity of staining was as: 0 (negative), 1 (weak), 2 (moderate) and 3 (strong). These two scores were then multiplied to form a staining index. In cases where the staining index was <4, it was classed as low expression, whereas in cases where the staining index was ≥4, it was classed as high expression of YAP1 or AGK.

### 2.4 Plasmid construction and cell transfection

To knock down the gene expression, we designed four different siRNAs, and a negative control, that is YAP1 siR‐1, 5′‐CUGCCACCAAGCUAGAUAATT‐3′; YAP1 siR‐2, 5′‐GGUGAUAUAUCAACCAAATT‐3′; AGK siR‐1, 5′‐GGAGGUUGUUACUGGUGUUTT‐3′; AGK siR‐2, 5′‐CCACCAUUGAACUGUCCAUTT‐3′; and a negative control siRNA, 5′‐UUCUCCGAACGUGUCACGUTT‐3′.

Furthermore, full‐length complementary DNA (cDNA) of human YAP1 with the accession number of NM_000011.00 and AGK with the accession number of NM_000007.14 were obtained using PCR amplification. These were then subcloned into a pcDNA3.1‐flag vector (Invitrogen, Carlsbad, CA, USA). An AGK‐luciferase reporter plasmid was also constructed by subcloning the human *AGK* proximal promoter region (−2000 bp to −1 bp) into a pGL3‐Basic vector (Promega, Madison, WI) at the MluI‐XhoI site, while a AGK promoter‐luciferase construct with a mutant TEAD‐binding site was generated using a QuikChange Site‐Directed Mutagenesis Kit (Stratagene‐Agilent, Santa Clara, CA, USA). The TEAD luciferase reporter was constructed by subcloning the 4 TEAD‐binding sequences (5ʹ‐CACATTCCTC‐3ʹ) into the pGL3‐Basic vector. All plasmids were then amplified in *E. coli* and DNA‐sequencing was confirmed before being used for cell transfection.

For siRNA transfection, cells were grown to 50%‐60% confluency and transfected with different siRNA constructs by using Lipofectamine 2000 (Invitrogen) according to the manufacturer's protocol. For gene transfection, cells were grown to reach 80%‐90% confluency and transfected with plasmids carrying YAP1 or AGK cDNA by using Lipofectamine 2000 for 48 or 72 hours. The efficiency of the knockdown or overexpression was then assayed by using qRT‐PCR and Western blot.

### 2.5 Immunofluorescence

Cells were seeded into a 6‐well plate and grown overnight to reach appropriate confluency and then transiently transfected with siAGK, Flag‐AGK plasmid and their corresponding negative controls for 48‐72 hours. At the end of each experiment, cells were washed with ice‐cold phosphate‐buffered saline (PBS) and fixed in 4% paraformaldehyde for 15 minutes, and then permeabilized in 0.2% Triton X‐100 (Roche Diagnostics Co., Indianapolis, IN, USA) for 15 minutes and blocked in 2% bovine serum albumin (BSA)/PBS at the room temperature for 60 minutes. The cells were subsequently incubated with a primary antibody, diluted with 0.1% BSA at 4°C overnight, and on the next day, the cells were washed three times with PBS and further incubated with a fluorescent dye‐labelled secondary antibody at room temperature for 45 minutes in the dark, and reviewed under a fluorescence microscope (Nikon, Tokyo, Japan).

### 2.6 Quantitative reverse transcriptase‐polymerase chain reaction (qRT‐PCR)

Total cellular RNA was isolated from cells using a TRIzol reagent (Invitrogen) and reversely transcribed into cDNA using the TransScript All‐in‐One First‐Strand cDNA Synthesis kit (TransGen Biotech, Beijing, China) according to the manufacturers’ protocol. qPCR was amplified in triplicate using the Fast Start Universal SYBR Green Master mix (Takara, Tokyo, Japan) according to the manufacturer's protocol in an ABI 7500 real‐time fast PCR system (Applied Biosystems, Waltham, MA, USA). Primer sequences of qPCR were YAP1, 5ʹ‐TCGTTTTGCCATGAACCAGA‐3ʹ and 5ʹ‐GGCTGCTTCACTGGAGCACT‐3ʹ; AGK, 5ʹ‐CCTGACACCATCAGCAAAGG‐3ʹ and 5ʹ‐CTCCGGGATAAGCAAAGTGC‐3ʹ; CYR61, 5ʹ‐CAGGACTGTGAAGATGCGGT‐3ʹ and 5ʹ‐GCCTGTAGAAGGGAAACGCT‐3ʹ; CTGF, 5ʹ‐GTGGAGTATGTACCGACGGC‐3ʹ and 5ʹ‐TCCGGGACAGTTGTAATGGC‐3ʹ; Survivin, 5ʹ‐TGCACCACTTCCAGGGTTTA‐3ʹ and 5ʹ‐AGAGAGAAGCAGCCACTGTT‐3ʹ; CDX‐2, 5ʹ‐CGGCAGCCAAGTGAAAAC‐3ʹ and 5ʹ‐GATGGTGATGTAGCGACTGTAGTG‐3ʹ; and GAPDH, 5ʹ‐CAGGGCTGCTTTTAACTCTGGT‐3ʹ and 5ʹ‐GATTTTGGAGGGATCTCGCT‐3ʹ. The melting temperature of qPCR was adjusted according to the melting temperature of each paired primer and was quantified using 2^−(Ct‐Cc)^ (Ct and Cc were the mean threshold cycle differences after normalizing to GAPDH).

### 2.7 Cell viability CCK‐8 assay

Gastric cancer cells were seeded into 96‐well plates and transfected with different genes for 48 hours. Cells were then subjected to a cell viability assay, comprising up to 120‐hour incubation. At the end of each experiment, the cell culture was combined with 10 μl of CCK‐8 solution (TransGen Biotech), further incubated for 4 hours, and the optical density value was measured by using a microplate reader (Thermo Scientific) at the absorbance at 450 nm. The experiments were performed in triplicate and repeated at least three times.

### 2.8 Tumour cell colony formation assay

Gastric cancer cells were seeded into 6‐cm dishes and transfected with different genes for 24 hours. Cells were then subjected to a colony formation assay, that is cells were trypsinized and re‐seeded into 6‐cm plates with a density of 1000 cells per well in triplicate and cultured for 14 days, throughout which time the medium was exchanged every three days. At the end of each experiment, cells were fixed with methanol and stained with 0.1% crystal violet solution and the numbers of cell colonies were counted.

### 2.9 Tumour cell invasion assay

Gastric cancer cells were seeded into 6‐cm dishes and transfected with different genes for 48 hours and then subjected to the Transwell invasion assay, that is Transwell with 8‐μm pore‐size filter (Merck KGaA, Darmstadt, Germany) was pre‐coated with 50‐μL Matrigel (BD Biosciences, San Jose, CA, USA). HGC‐27 (1 × 10^5^ cells) or BGC‐823 (1 × 10^5^ cells) were added into the upper chamber, while 600 μL of DMEM containing 15% FBS was added to the low cell culture well and incubated for 24 hours. At the end of each experiment, cells that invaded into the lower surface of the filter were fixed, stained and counted. The experiments were performed in duplicate and repeated at least once.

### 2.10 Nude mouse tumour cell xenograft assay

The animal protocol of this study was approved by the Institutional Animal Care and Use Committee (IACUC) of The First Affiliated Hospital to Nanchang University (Nanchang, China). In this study, BALB/c nude mice at four weeks of age were purchased from the Center of Experimental Animal of Guangzhou University of Chinese Medicine (Guangzhou, China) and randomly assigned to two groups (n = 6). Scramble short hairpin RNAs or short hairpin RNAs targeting AGK were subcloned into the lentiviral expression vector, GV248 (Genepharma). Animals were then subcutaneously injected with 2 × 10^6^ of HGC‐27 cells that were stably expressing LV‐shAGK or LV‐scramble shRNA. Growth of the tumour cell xenografts was monitored daily and two‐dimensional measurements were made using electronic digital calipers (Thermo Scientific) at the indicated period of time (Figure 2E). Tumour volume was calculated by using the formula of 3.14/6 × *L* × *W* ^2^ (*L* = tumour length, *W* = tumour width). After 31 days, the mice were killed by euthanasia and tumour xenografts were harvested and weighed.

### 2.11 Luciferase reporter assay

HEK‐293T cells were seeded into 24‐well plates at a density of 2 × 10^6^/well in triplicate, grown overnight and transfected with the indicated plasmids using a Lipofectamine 2000 reagent (Invitrogen), according to the manufacturer's instructions for 48 hours. After that, the luciferase activity was measured by using the Dual‐Luciferase Reporter Assay System (Promega), according to the manufacturer's protocol.

### 2.12 Chromatin immunoprecipitation (ChIP) assay

HCG‐27 cells were grown and transfected with different plasmids and then harvested for chromatin purification. The cells were then subjected to immunoprecipitation with an anti‐YAP (#14074, Cell Signaling Technology) or anti‐IgG as a negative control (#3900, Cell Signaling Technology). The ChIP assay was conducted using the Chromatin Immunoprecipitation (ChIP) Assay Kit (Millipore Corporation), according to the manufacturer's protocol. The resulting DNA samples were subjected to PCR amplification of the putative TEAD‐binding site‐containing fragment in the promoter area of AGK with the following primers: (5′‐GAGTGTCAGGTTTCTGTT‐3′ and 5′‐AGGTTCCCAGTCTGAGTC‐3′, resulting in a 292 bp product, and 5′‐CAGAGTGAGAACCCTGTC‐3′ and 5′‐CTGACCAAAGGTAAAGTG‐3′, resulting in a 216 bp product).

### 2.13 Statistical analysis

The data were statistically analysed using the SPSS 18.0 software (SPSS, Chicago, IL, USA). The association of the overall survival of patients with AGK expression was plotted with Kaplan‐Meier curves and statistically analysed with the log‐rank test. The association of AGK expression with clinicopathological parameters from patients was assessed by using Pearson's chi‐square test or Fisher's exact test. The correlation between AGK and YAP1 expressions was analysed with a Spearman rank correlation test. Data on qRT‐PCR, colony formation, cell invasion and luciferase reporter assays were compared by using Student's *t* test. A multi‐way classification analysis of variance test was performed to statistically analyse data on the cell viability and tumour cell xenograft assays. A two‐sided tested *P*‐value of <.05 was considered to be statistically significant.

## 3 RESULTS

### 3.1 Up‐regulation of AGK protein in gastric cancer tissues and cell lines, and the association with clinicopathological features of patients

**FIGURE 1:**
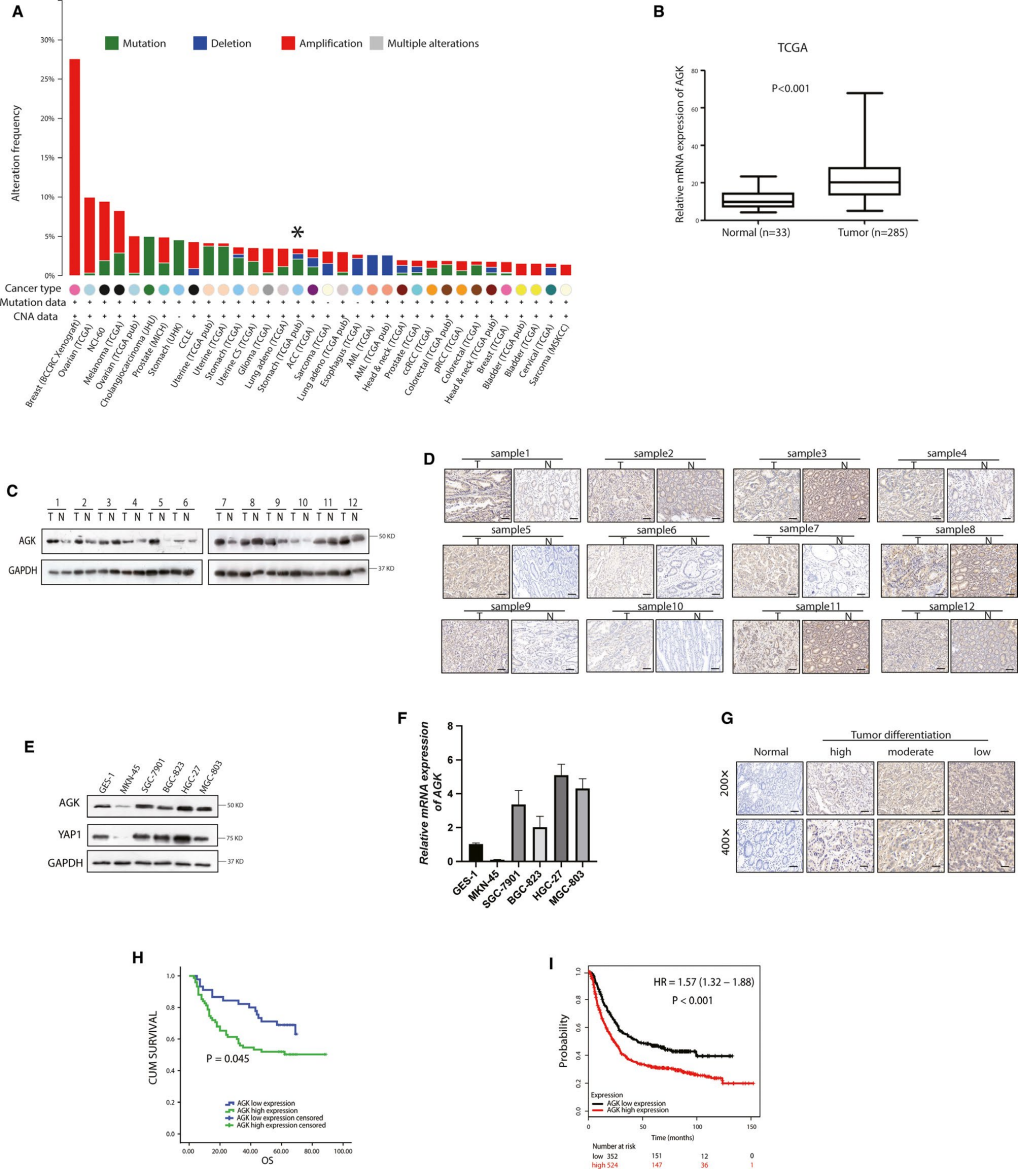
AGK protein up‐regulation in gastric cancer tissues and the association with clinicopathological parameters from patients. A, Analysis of the TCGA data. The alteration frequency of AGK in various cancer tissues. The data were modified from the cBioPortal for Cancer Genomics (http://www.cbioportal.org/). B, Analysis of the TCGA data. Expression of AGK mRNA was significantly higher in gastric cancer tissues (n = 285) than that in normal tissues (n = 33). C and D, Western blot and immunohistochemistry results of AGK protein levels in GC tissues and adjacent non‐cancerous gastric tissues. AGK protein levels are higher in GC tissues (T) than in adjacent normal gastric tissues (N) from the same patient (n = 12). E and F, An immortal gastric epithelial cell line (GES‐1) and five gastric cancer cell lines (MKN‐45, HGC‐27, SGC‐7901, BGC‐823 and MGC‐803) were grown and subjected to protein and mRNA extraction for AGK and YAP1. G, Immunohistochemical staining of AGK in the formalin‐fixed, paraffin‐embedded gastric cancer tissues and normal gastric tissues. H, Kaplan‐Meier curves stratified by AGK expression in 120 cases of gastric cancer patients. I, Kaplan‐Meier curve stratified by AGK expression in gastric cancer patients obtained from the TCGA‐STAD database

In this study, we first assessed AGK expression using online database data and found that AGK was not significantly amplified in gastric cancer on the TCGA database (http://www.cbioportal.org/) data (Figure 1A). However, the TCGA‐STAD database data showed that the level of AGK mRNA was dramatically higher in gastric cancer tissue than in normal tissues (Figure 1B). Our own Western blot and immunohistochemistry data show that the expression of the AGK protein was higher in gastric cancer tissues than in the paired non‐cancerous tissues (Figure 1C,D). In addition, we compared the levels of AGK mRNA and protein expression in five gastric cancer cell lines (MKN‐45, HGC‐27, SGC‐7901, BGC‐823 and MGC‐803) and one immortal gastric epithelial cell line (GES‐1). Interestingly, we found that the AGK mRNA and protein levels were low in YAP1 deficient MKN‐45 cells, while YAP1‐expressing gastric cancer cell lines had higher AGK expression compared with GES‐1 cells (Figure 1E,F). These data indicate the oncogenic role of AGK and an association of AGK with YAP1 in gastric cancer.

In the current study, the AGK expression was analysed in the tissue samples of 120 cases of gastric cancer using immunohistochemistry (Figure 1G). We then associated AGK expression with clinicopathological data from patients and found that AGK expression was significantly associated with histological differentiation (*P* = .009), but not with other clinicopathological data (Table S1). The Kaplan‐Meier curves and the log‐rank analysis revealed that the overall survival was lower in patients with high AGK‐expressing tumours than in the patients with low AGK‐expressing tumours l (53.8 ± 4.2 months vs 56.7 ± 3.2 months; *P* = .045; Figure 1H). Furthermore, bioinformatic analysis of the online KM plotter database data (kmplot.com/analysis/index.php?p=service&cancer=gastric) supported our findings (Figure 1I).

### 3.2 AGK promotion of gastric cancer cell proliferation in vitro and in vivo

**FIGURE 2:**
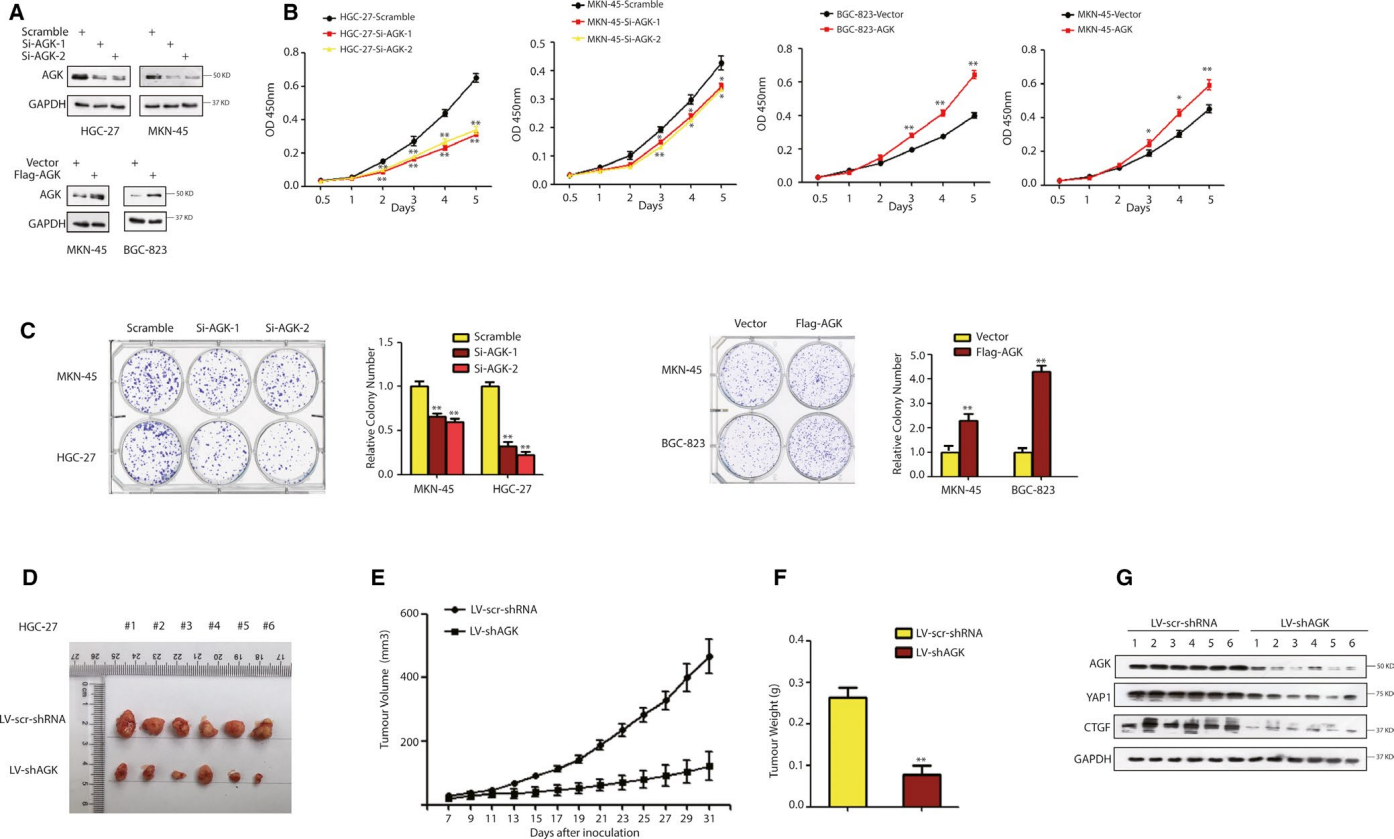
AGK promotion of gastric cancer cell proliferation in vitro and in vivo. A, Cells were transfected with AGK siRNAs, cDNA or the negative control, and then subjected to Western blot. The efficiency of AGK knockdown and overexpression in HGC‐27, BGC‐823 and MKN‐45 cells was determined. GAPDH was used as a loading control. B, Cells were transfected with AGK siRNAs, cDNA or the negative control, and then subjected to CCK‐8 assay. Overexpression of AGK in BGC‐823 and MKN‐45 cells promoted cell proliferation rate, whereas knockdown of AGK in HGC‐27 and MKN‐45 cell inhibited cell proliferation rate, less significantly in YAP1‐deficient (MKN‐45) cells. C, Cells were transfected with AGK siRNAs, cDNA or the negative control, and then subjected to colony formation assay. Knockdown of AGK inhibits clonogenic ability of gastric cancer cells, overexpression of AGK promotes clonogenic ability of gastric cancer cells, less significantly in YAP1‐deficient (MKN‐45) cells. D‐G, Nude mouse tumour cell xenograft assay. D, The images of xenograft tumours that were harvest at the end of experiments. E, Growth curves of xenograft tumours derived from HGC‐27 cells that expressed LV‐scramble‐shRNA or LV‐shAGK. F, Comparison of the average weight of collected tumours from above two groups. G, The protein levels of AGK, YAP1 and CTGF were detected in six tumour samples by WB analysis with indicated antibodies. The data are summarized as the mean ± SD of three independent experiments. *P < .05, **P < .01 by two‐tailed *t* test

We then assessed the effect of AGK overexpression and knockdown on gastric cancer viability. Based on the expression levels of AGK in gastric cancer cell lines, BGC‐823 and HGC‐27 cells were chosen for overexpression and knockdown assays, respectively. CCK‐8 assay data show that the rate of BGC‐823 cell proliferation was significantly higher in AGK‐overexpressing tumour cells than in the control, whereas the proliferation of HGC‐27 cells was reduced after knockdown of AGK expression (Figure 2A,B). Furthermore, a colony formation assay revealed the same trends in the BGC‐823 and HGC‐27 cell lines, respectively (Figure 2C). Our nude mouse tumour cell xenograft assay further demonstrated that knockdown of AGK expression in HGC‐27 cells resulted in a lower growth rate and the average volume of tumour cell xenografts than in scramble shRNA‐transfected cells (Figure 2D‐F). However, we repeated our experiments in YAP1‐deficient MKN‐45 cells and found that, in these cells, AGK only mildly regulated tumour cell proliferation (Figure 2B,C). This indicated that YAP1 might play important roles in the mediation of the effects of AGK in gastric cancer cells. To test our cell‐based findings, we performed WB analysis using the xenograft tumours. Interestingly, the protein levels of YAP1 and its target CTGF^7^ were down‐regulated in LV‐shAGK group (Figure 2G), which further suggested that AGK depletion retards tumour growth by mainly inactivating YAP.

### 3.3 AGK promotion of gastric cancer cell invasion and epithelial‐mesenchymal transition

**FIGURE 3:**
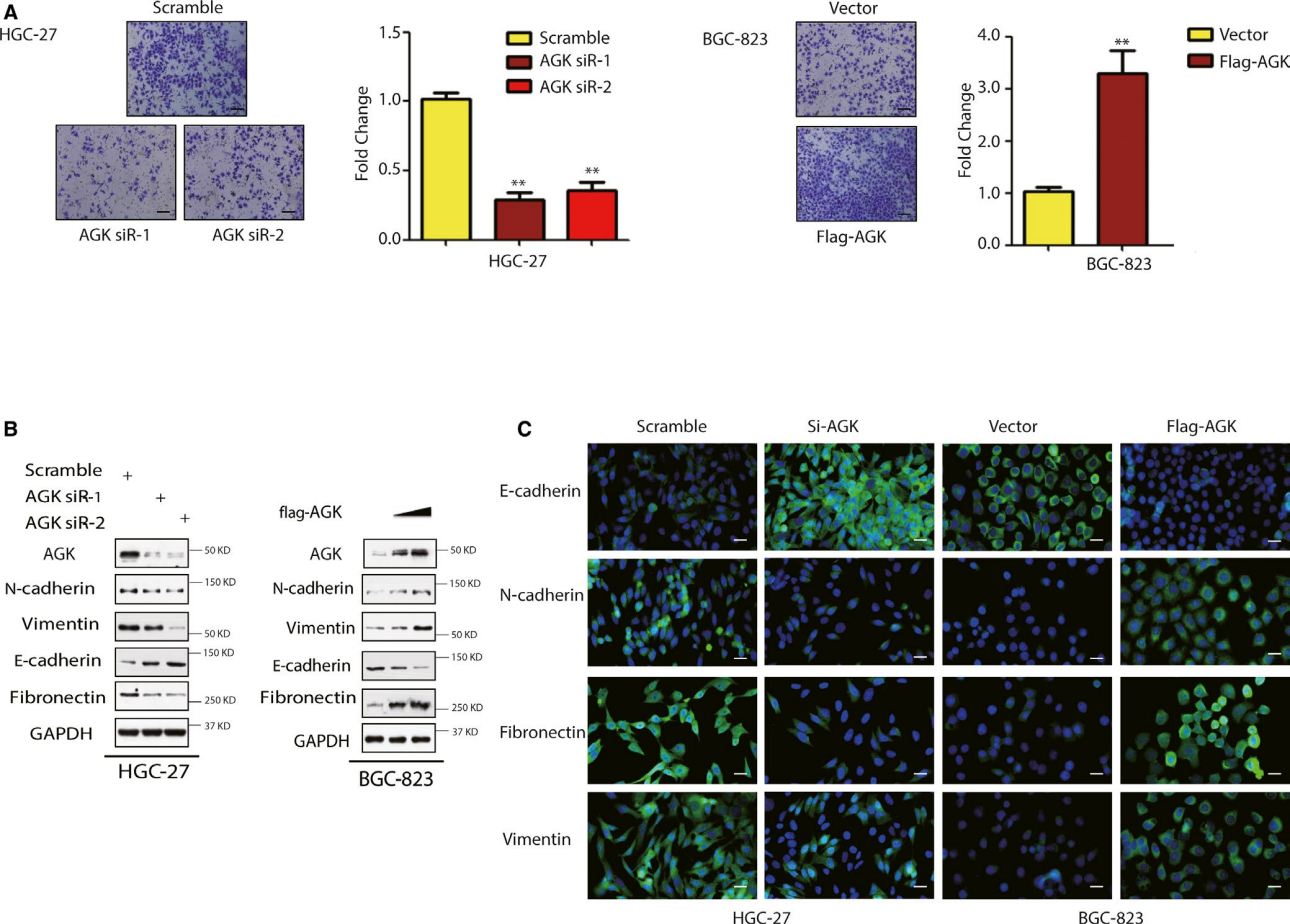
AGK promotion of gastric cancer cell invasion and epithelial‐mesenchymal transition. A, Cells were transfected with AGK siRNAs, cDNA or the negative control, and then subjected to Transwell invasion assay. Knockdown of AGK inhibits invasion ability of HGC‐27 cells, overexpression of AGK promotes invasion ability of BGC‐823 cells. B, Cells were transfected with AGK siRNAs, cDNA or the negative control, and then subjected to Western blot to evaluate the expression of AGK, N‐cadherin, vimentin, E‐cadherin and fibronectin. Knockdown of AGK increased E‐cadherin expression and inhibited N‐cadherin, fibronectin and vimentin protein expression in HGC‐27 cells. Conversely, overexpressing AGK led to opposite results in BGC‐823 cells. C, Immunofluorescence results for EMT markers after GC cells were transfected with AGK siRNA or cDNA. The data are summarized as the mean ± SD of three independent experiments. *P < .05

Next, we determined the effect of AGK on the gastric cancer invasion capacity using a tumour cell invasion assay. Down‐regulation of AGK expression, using AGK siRNA, significantly lowered the capacity for HGC‐27 cell invasion. Furthermore, AGK overexpression induced BGC‐823 cell invasion (Figure 3A). At the gene level, manipulation of AGK expression also altered the expression of epithelial‐mesenchymal transition (EMT)‐related proteins. For example, AGK‐overexpressing BGC‐823 cells showed a higher expression level of N‐cadherin, vimentin and fibronectin than control cells, but a lower expression level of E‐cadherin than control cells (Figure 3B,C). In contrast, knockdown of AGK expression had the opposite results in HGC‐27 cells (Figure 3B,C).

### 3.4 Association of YAP1 and AGK expression in gastric cancer ex vivo and in vitro

**FIGURE 4:**
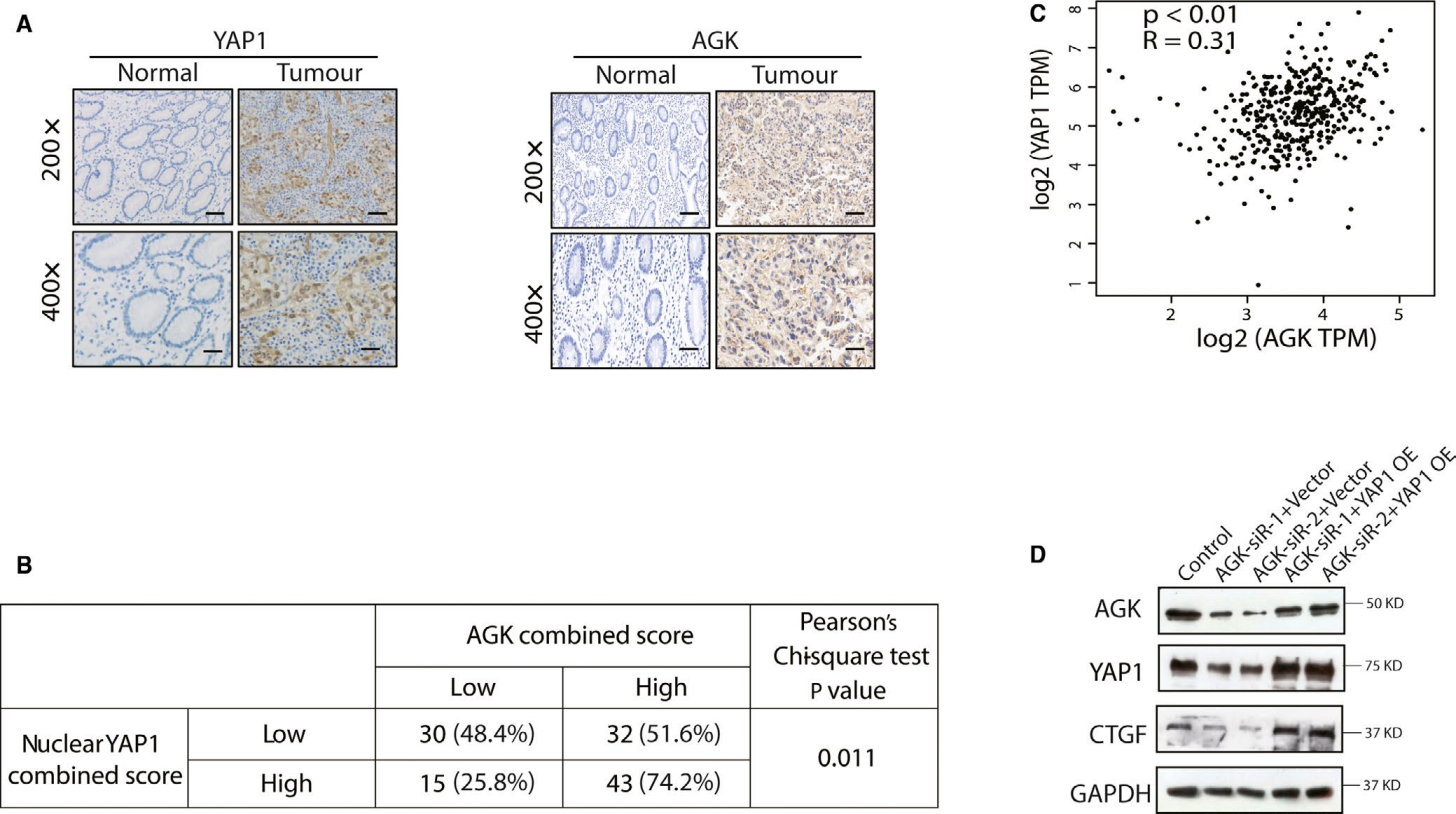
Association of YAP1 with AGK proteins in gastric cancer cells and tissues. A, The expression of YAP1 with AGK protein in gastric cancer tissues is shown in two representative cases. B, The correlation of staining intensity between YAP1 and AGK in gastric cancer tissues. C, Association of YAP1 with AGK expression in gastric cancer tissues from the TCGA‐STAD database data, plotted in the GEPIA website. D, The protein expressions of AGK, YAP1 and CTGF were evaluated in HGC‐27 cells that were transfected with non‐target siRNA + pcDNA3.1 vector, AGK siRNAs + pcDNA3.1 vector or the combination of AGK siRNAs and YAP cDNA

Aberrant YAP1 and AGK expressions have been reported in various human cancers. Our current data indicate an association of AGK with YAP1 in gastric cancer; thus, we compared the expression of these proteins in 120 gastric cancer tissue samples and found that both YAP1 and AGK were significantly up‐regulated in tumour tissues, which is consistent with previous studies.^15^, ^26^, ^27^ YAP1 staining was mainly observed in the nuclei with little staining in the cytoplasm of tumour cells, whereas AGK was only observed in the cytoplasm of tumour cells (Figure 4A). The Pearson chi‐square test result shows that the nuclear YAP1 expression was highly associated with cytoplasmic AGK expression (Figure 4B). In addition, the TCGA database data (http://gepia.cancer‐pku.cn/detail.php?clicktag=correlation) further confirmed this finding, showing that AGK expression was associated with YAP1 expression in gastric cancer tissues (Figure 4C). Additionally, we found that knockdown of AGK decreased the level of CTGF, and this effect was reversed by overexpression of YAP1. We also noticed that transfection of YAP1 increases AGK protein level (Figure 4D). This Western blot result and the observation that YAP1 and AGK were up‐regulated and highly associated in GC cell lines and tissues, raising that there might be a reciprocal interplay between YAP1 and AGK.

### 3.5 YAP1 transcriptional up‐regulation of AGK expression in gastric cancer cells

**FIGURE 5:**
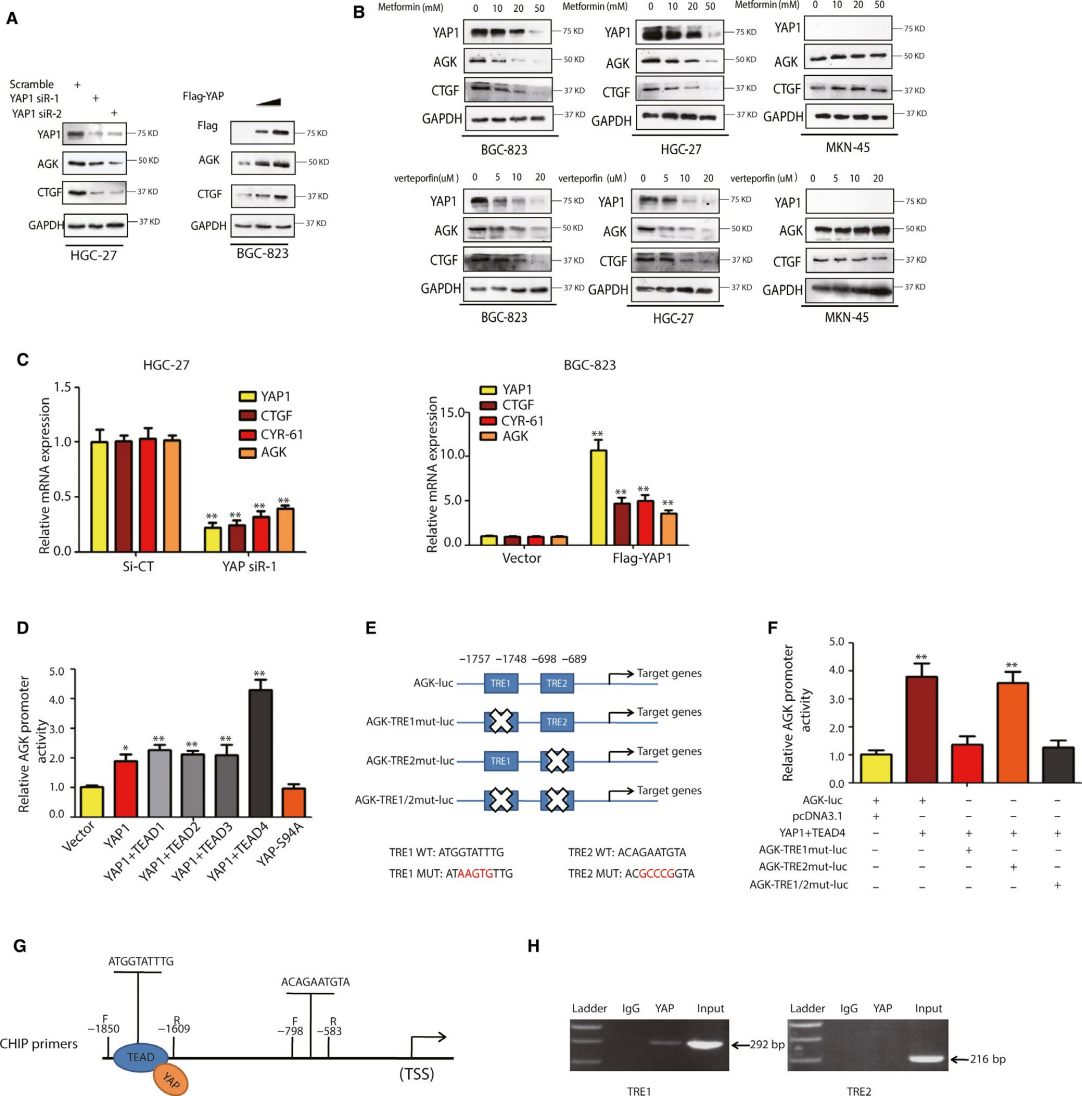
YAP1 transcriptional regulation of AGK expression in gastric cancer cells. A, The levels of AGK and CTGF proteins were analysed by using Western blot in YAP‐siRNA or cDNA‐transfected HGC‐27 and BGC‐823 cells. B, HGC‐27, BGC‐823 and MKN‐45 cells were treated with metformin (0, 10, 20 or 50 mmol L^−1^) and verteporfin (0, 5, 10 or 20 μmol L^−1^) for 48 h and analysed for YAP1, AGK and CTGF expression. C, qRT‐PCR results indicated that after knocking down YAP1, AGK along with Hippo‐YAP1 target gene mRNAs, such as for CTGF and CYR61, were decreased in HGC‐27 cells, while overexpressing YAP1 increased AGK, CTGF and CYR61 mRNA levels in BGC‐823 cells. D, AGK promoter activity was detected in HEK‐293T cells that transfected with YAP1 cDNA, YAP‐S94A or the combination of YAP1 and TEAD1‐4 cDNA. E, Illustration of luciferase reporter constructs (wild‐type and mutated‐type). Mutant luciferase constructs were generated by site‐directed mutagenesis of TRE1 or TRE2 alone or in combination. F, HEK293T cells were grown and cotransfected with the indicated plasmids and then subjected to luciferase reporter assay. The mutated TRE1 in the AGK promoter was sufficient to abolish AGK promoter activity induced by YAP/TEAD4 cDNA. G, Design of the AGK promoter primers for ChIP assays. H, Chromatin from HGC‐27 was pulled down using a YAP1 antibody or IgG. PCR amplification using TRE1 or TRE2 primers was then performed. A 292 bp PCR product containing TRE1 in the AGK promoter was amplified from the chromatin DNA that was pulled down by the YAP antibody, while the input chromatin is shown as a positive control for ChIP assay

To further confirm the role of YAP1 in the regulation of AGK in gastric cancer cells, we associated AGK expression with other YAP1‐related proteins in gastric cancer tissue samples and found that AGK expression was also associated with the expression of TEAD1, TEAD2 and TEAD4 (Figure S1A). Thus, we knocked down or overexpressed YAP1 using YAP1 siRNA and cDNA, respectively, and found that knockdown or overexpression of YAP1 in BGC‐823 and HGC‐27 cells changed the expression of AGK and CTGF proteins, respectively (Figure 5A). Furthermore, we treated gastric cancer cells with verteporfin or metformin, the YAP inhibitors,^28^ for 48 hours, and found that verteporfin and metformin dose‐dependently down‐regulated the level of AGK protein in BGC‐823 and HGC‐27 cells. However, treatment of YAP1‐deficient MKN‐45 cells with metformin and verteporfin resulted in no significant change in the AGK protein expression level (Figure 5B). Similar results were observed at the mRNA level of YAP1 and AGK in these cell lines, for which CTGF and CYR61 were used as positive controls (Figure 5C).

Next, we assessed the underlying molecular events that might be involved in YAP1 regulation of AGK expression by using luciferase reporter assays. Our data show that YAP1 induced the luciferase activity of the AGK promoter, while cotransfection of YAP1 and each TEAD family member (TEAD1‐4) further enhanced the luciferase activity of the AGK promoter compared with the results when the cells were transfected with YAP1 only (Figure 5D). The YAP1 + TEAD4 group showed the strongest induction of luciferase activity of all of the groups that were tested (Figure 5D). This is consistent with the results of a previous study that identified TEAD4 as the major YAP1 interacting TEAD family member.^29^ Furthermore, we also assessed whether the YAP1/TEADs interaction is essential for AGK transcription by generating a mutated YAP‐S94A that lacks TEAD‐binding capacity. Our data show that this mutated YAP‐S94A failed to activate the luciferase activity of the AGK promoter (Figure 5D). Taken together, these results suggest that AGK could be a transcriptional target of YAP1/TEADs.

To further map the TEAD response element (TRE) that is responsible for TEAD binding and AGK promoter activation, we found two potential TREs (TRE1 and TRE2) and mutated them individually (Figure 5E). The luciferase reporter assay used these mutated constructs after cotransfection with YAP1 and TEAD4 and showed that TRE1 mutation was sufficient to abolish AGK promoter activity (Figure 5F). Furthermore, we performed a chromatin immunoprecipitation assay to further validate the binding of TEAD to the AGK promoter and found that YAP1/TEADs was able to bind to TRE1 in the AGK promoter, but not TRE2 to induce AGK transcription (Figure 5G,H, and Figure S1B).

### 3.6 AGK inhibits the Hippo‐YAP1 pathway and up‐regulates TEAD transcriptional activity

**FIGURE 6:**
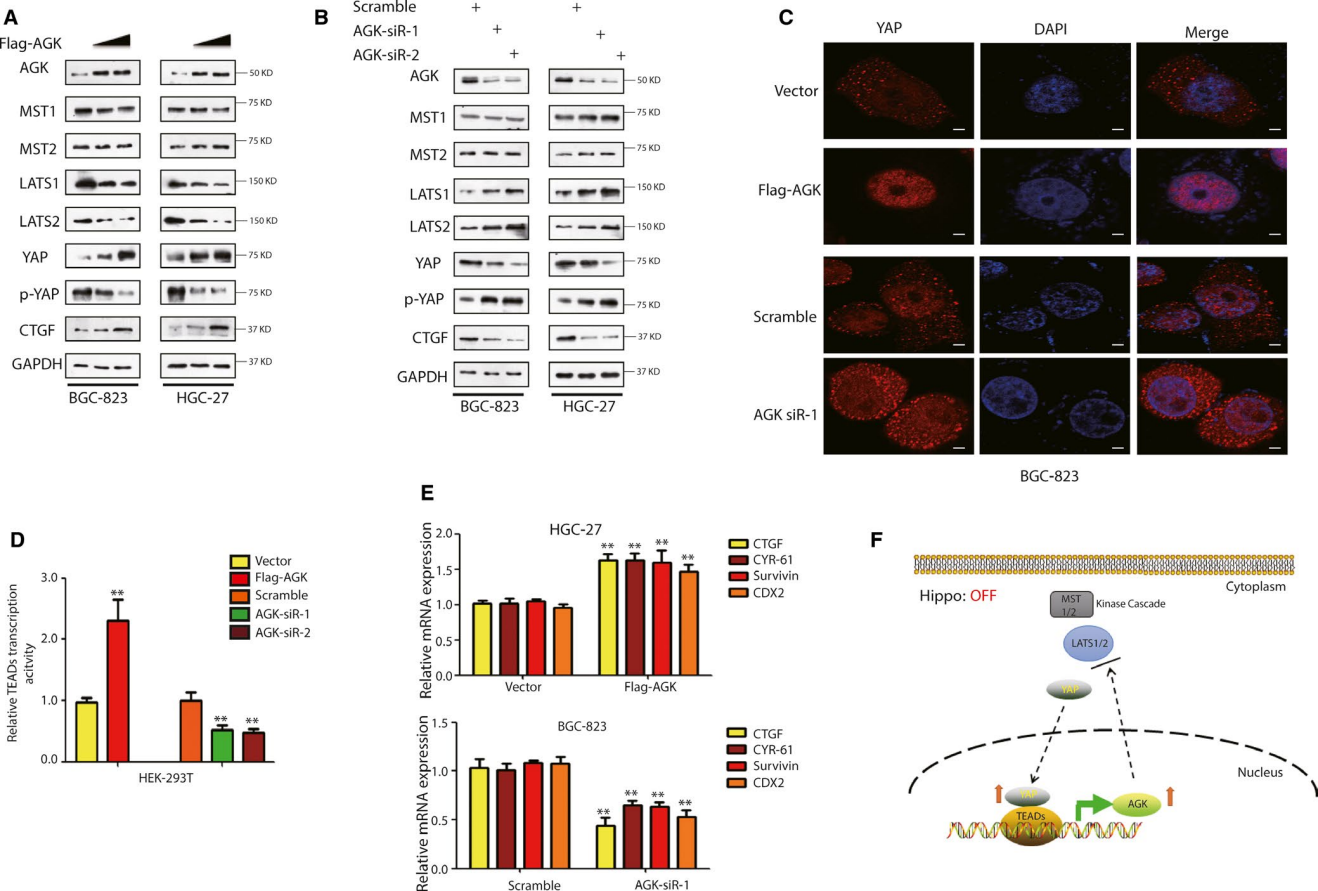
AGK inhibition of the Hippo‐YAP1 pathway and up‐regulation of TEAD transcriptional activity. A, B, Western blot analysis of MST1/2, LATS1/2, p‐YAP, YAP1 and CTGF expression after transfection with AGK siRNAs (A) and plasmids carrying AGK cDNA (B) in HGC‐27 and BGC‐823 cells, respectively. C, Confocal images of YAP1 staining in BGC‐823 cells after transfection with AGK siRNA or AGK cDNA. D, Relative TEAD luciferase reporter activity in AGK after transfection with AGK siRNA or negative control siRNA. E, AGK overexpression resulted in an increase in the expression of Hippo‐YAP/TEAD target genes, including CTGF, CYR61, survivin and CDX2. Conversely, knockdown of AGK had the opposite effect on expression of these genes. F, Illustration of the YAP1‐AGK positive feedback loop in gastric cancer cells

We then assessed the interaction of AGK and Hippo‐YAP1 pathway in gastric cancer cells and found that AGK overexpression was able to up‐regulate level of YAP1 and CTGF proteins, but lead to a down‐regulation of the level of LATS1/2 and p‐YAP without changing in MST1/2 levels (Figure 6A). Furthermore, knockdown of AGK expression had the reverse results (Figure 6B). Given the association of AGK with the Hippo‐YAP1 pathway, we speculated whether the YAP1 level was determined by the AGK expression. Thus, we overexpressed and knocked down the expression level of AGK in BGC‐823 cells and found that AGK overexpression promoted nuclear YAP1 accumulation, whereas knockdown of AGK expression induced cytoplasmic YAP1 presence (Figure 6C). Furthermore, because TEADs are major transcriptional factor partners for YAP1, we examined whether AGK regulated the transcription activity of TEADs in vitro. Because all of the members of the TEAD family of proteins can bind to the consensus sequences of MCAT (5ʹ‐CATTCCA/T‐3ʹ) in the evolutionarily conserved DNA binding domain,^30^ the TEAD‐binding site of 5ʹ‐CACATTCCTC‐3ʹ was subcloned into the luciferase reporter and then transfected to HEK‐293T cells for the luciferase reporter assay. Our data show that AGK expression was associated with the level of the transcription activity of TEADs (Figure 6D). Our qRT‐PCR data further confirmed this finding (Figure 6E), that is knockdown of AGK expression reduced the expression of some of the YAP1‐TEADs target mRNAs, such as CTGF, CYR61, survivin and CDX2. Furthermore, AGK overexpression increased the level of these mRNAs (Figure 6E). Taken together, our data indicate that AGK is able to up‐regulate the expression of the Hippo‐YAP1 pathway downstream genes through the enhancement of the transcriptional activity of TEADs. Our findings suggest that AGK is not only a novel target of the Hippo‐YAP1/TEADs pathway but also a repressor of the Hippo pathway; thus, acting as a stimulator of the transcription activity of YAP1/TEADs to form a positive feedback circuit in gastric cancer cells (Figure 6F).

## 4 DISCUSSION

The abnormal expression or dysregulation of multiple signalling pathway genes contributes to gastric cancer development and progression.^31^ Although there have been recent advancements in the early detection and treatment of gastric cancer, most patients are still suffering from drug resistance and tumour recurrence, leading to high levels of morbidity and mortality worldwide. Thus, the improvement of our knowledge of the molecular mechanisms of gastric tumorigenesis and cancer progression would have a significant impact on early detection, prediction of prognosis and novel therapeutic strategies for gastric cancer. Towards this end, our current study focused on AGK and the related gene pathways. Previous studies have shown that AGK is overexpressed in various types of human cancer and is associated with tumour cell proliferation and tumorigenicity.^20^, ^21^, ^22^, ^23^ In our current study, we found that the AGK protein was significantly up‐regulated in gastric cancer cell lines and tissue samples. Ectopic AGK expression induced gastric cancer cell proliferation and epithelial‐mesenchymal transition in vitro. However, knockdown of AGK expression showed the opposite results in gastric cancer cells in vitro and in nude mouse tumour cell xenograft growth. We also found that AGK expression was associated with tumour cell de‐differentiation and poor overall survival of gastric cancer patients. Future studies will further evaluate whether detection of AGK expression serves as a marker for early gastric cancer diagnosis or prediction of prognosis.

Indeed, several previous studies have demonstrated an oncogenic role of AGK in various types of human cancer. For example, AGK expression induces breast cancer cell proliferation and enhances the G_1_‐S phase transition. Furthermore, at the protein level, AGK is able to activate PI3K/AKT/FOXO signalling, down‐regulate the expression of cyclin‐dependent kinase inhibitors p21 and p27 and up‐regulate the expression of the cell cycle regulator cyclin D1.^22^ AGK expression also enhances hepatocellular cancer angiogenesis and inhibits tumour cell apoptosis through the activation of NF‐κB signalling.^21^ Moreover, the AGK protein is able to directly interact with the JH2 domain to restore JAK2 inhibition and activation of the JAK2/STAT3 signalling, leading to the promotion of the tumorigenicity of oesophageal squamous cell carcinoma cells.^23^ Taken together, these results suggest that AGK functions as an oncogene in these human cancers, including gastric cancer. At the molecular level, the effect of AGK might link to a suppression of the Hippo pathway and a subsequent boost of YAP1/TEADs transcription activity in gastric cancer cells. As we know, a lot of regulatory factors and cellular processes have been covered that can act on Hippo pathway, such as cell polarity, cell adhesion, cell stress, extracellular ligands and so on.^32^ Our current data are novel, and there have been no previous reports on the association of AGK with the Hippo pathway.

In cells, the YAP1 protein can form a protein complex with TEADs, which contains a DNA binding domain to bind to DNA for transcriptional regulation of gene expression for cell proliferation and wound healing, as well as for cancer development and progression.^33^, ^34^ Our current study further demonstrated that the expression of YAP1/TEADs and the AGK protein was highly associated and that YAP1/TEADs can transcriptionally up‐regulate AGK expression in gastric cancer cells. Our luciferase reporter assay results show that YAP1/TEADs proteins bind to the AGK gene promoter, but not to the mutated AGK gene promoter. These binding sites were localized at −1757 to −1748 nt (5ʹATGGTATTTG‐3ʹ) and −698 to −689 (5ʹ‐ACAGAATGTA‐3ʹ) of the AGK gene. Our Western blot analysis revealed that up‐regulated AGK expression led to a reduction of LATS1/2 and phosphorylated YAP at S127, although it rescued the level of total YAP1 protein, whereas knockdown of AGK expression had the opposite effects. These data indicate an interaction of AGK with YAP1/TEADs proteins in the regulation of gastric cancer cell proliferation and tumour progression. Furthermore, our immunofluorescence data localized YAP1 proteins in gastric cancer cells after knockdown or overexpression of AGK, that is there was an increase in the expression of nuclear YAP1 protein in AGK‐overexpressing cells, indicating that the YAP1 protein is activated in gastric cancer cells after AGK overexpression. Indeed, our luciferase reporter assay results show that AGK was able to induce the transcription activity of YAP1/TEADs genes. Previous studies have reported that LPA is a small molecular YAP1 activator and is able to induce LATS expression.^35^, ^36^ Intracellular LPA is generated by AGK phosphorylation of monoacylglycerol; however, our current data did not observe any restoration of YAP or LATS expression in the cells in which AGK was knocked down following treatment with LPA (Figure S1C), indicating that AGK activity might function differently from YAP1 expression. Moreover, our current data show that AGK overexpression reduced the half‐life of LATS1/2 proteins (Figure S1D), indicating a post‐transcriptional regulation of LATS1/2 protein in the Hippo‐YAP1–related gene signalling. Now, our group is further investigating the regulation and functions of AGK and YAP1 gene pathways in gastric cancer development and progression. In conclusion, our current study provides the first evidence that AGK expression is up‐regulated in gastric cancer cells and tissues, and that the up‐regulation of AGK is associated with poor overall survival of gastric cancer patients. We also demonstrated that AGK is a novel transcription target gene of YAP1/TEADs and is able to induce YAP1/TEADs transcription activity through the inactivation of the Hippo pathway to provide a positive feedback loop of YAP1 interaction with AGK in gastric cancer cells. Additionally, our study also delineated the feedback regulation between AGK and the Hippo pathway, which will contribute to a better understanding of the molecular mechanisms of AGK functions in gastric cancer. Future studies should assess whether the targeting of AGK expression or activity could be a therapeutic target for the control of gastric cancer progression.

## CONFLICTS OF INTEREST

The authors declare that they have no conflict of interest.

## AUTHOR CONTRIBUTION

**Shanshan Huang:** Data curation (lead); Validation (equal); Writing‐original draft (equal); Writing‐review & editing (equal). **Yuan Cao:** Investigation (equal); Methodology (equal); Validation (equal). **Hui Guo:** Data curation (equal); Investigation (equal); Methodology (equal); Validation (equal). **Yangyang Yao:** Formal analysis (equal); Methodology (equal). **Li Li:** Data curation (equal); Investigation (equal); Methodology (equal). **Jun Chen:** Data curation (equal); Validation (equal). **Junhe Li:** Conceptualization (equal); Software (equal); Supervision (equal). **Xiaojun Xiang:** Conceptualization (equal); Funding acquisition (equal); Supervision (equal). **Jun Deng:** Conceptualization (equal); Data curation (equal); Funding acquisition (equal); Supervision (equal); Writing‐review & editing (equal). **Jianping Xiong:** Conceptualization (equal); Data curation (equal); Funding acquisition (equal); Supervision (equal); Writing‐review & editing (equal).

## Supporting information

Figure S1

Table S1

## Data Availability

The data that support the findings of this study are available from the corresponding author upon reasonable request.
