# Supplementary figures and images for: Up‐regulated acylglycerol kinase (AGK) expression associates with gastric cancer progression through the formation of a novel YAP1‐AGK–positive loop
Source: J Cell Mol Med. 2020 Aug 22;24(19):11133–45. doi: 10.1111/jcmm.15613 (PMC7576242; doi:10.1111/jcmm.15613)

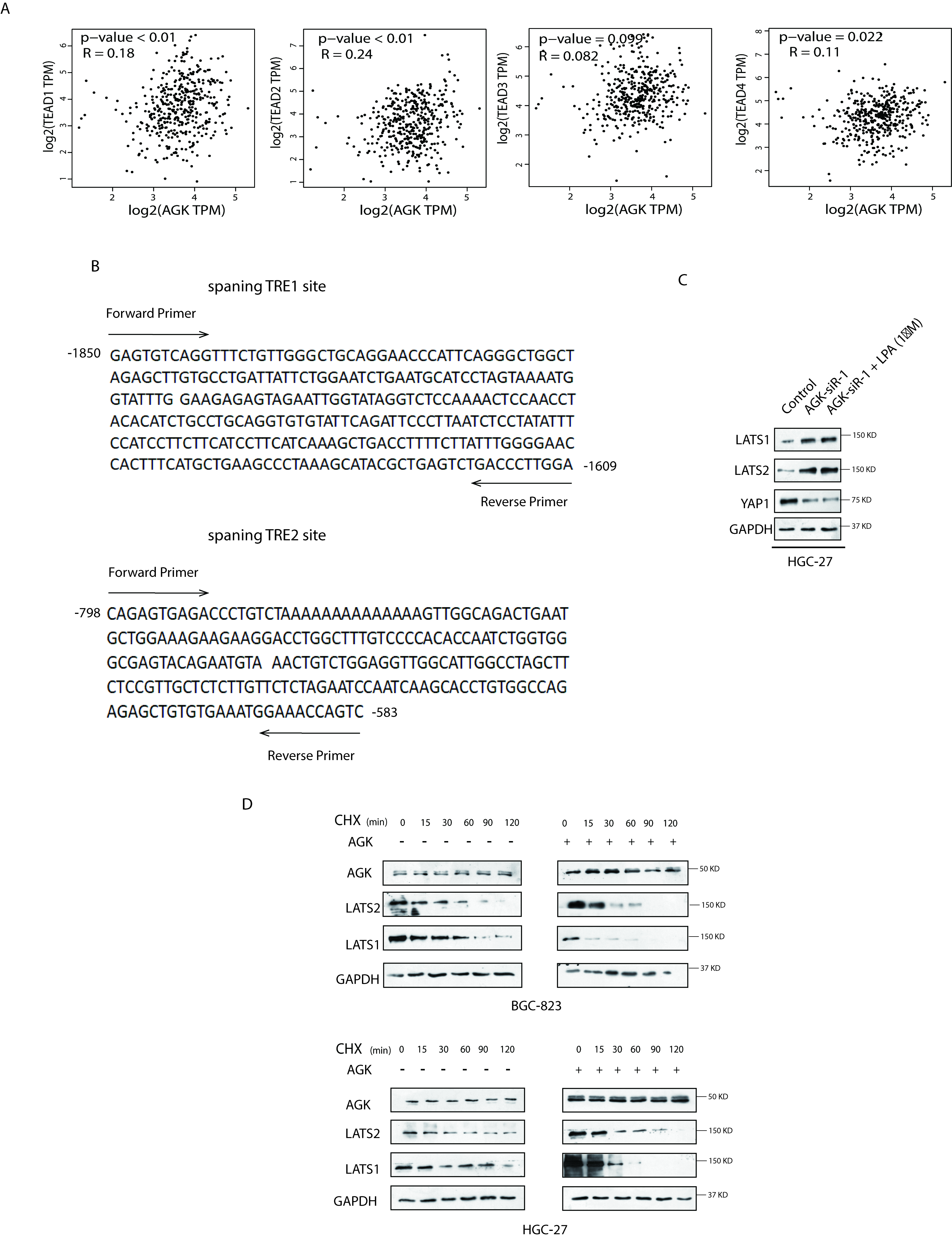

Supplement: Supplementary file 1 — Figure S1 [file JCMM-24-11133-s001.tif]
