# Supplementary material for: Up‐regulated acylglycerol kinase (AGK) expression associates with gastric cancer progression through the formation of a novel YAP1‐AGK–positive loop
Source: J Cell Mol Med. 2020 Aug 22;24(19):11133–45. doi: 10.1111/jcmm.15613 (PMC7576242; doi:10.1111/jcmm.15613)
Supplement: Supplementary file 2 — Table S1 [file JCMM-24-11133-s002.doc]

**Table 1: Relationship between AGK expression and clinicopathological variables (n=120).**

|  |  | **AGK expression** | | **P value** |
| --- | --- | --- | --- | --- |
| **Variables** | **Number** | **High** | **Low** |  |
| **Gender** |  |  |  | 0.792 |
| Male | 87 | 55(63.2) | 32(36.8) |  |
| Female | 33 | 20(60.6) | 13(39.4) |  |
| **Age^*^** |  |  |  | 0.346 |
| >57.5 | 60 | 35(58.3) | 25(41.7) |  |
| <57.5 | 60 | 40(66.7) | 20(33.3) |  |
| | **Depth of invasion** | | --- | |  |  |  | 0.612 |
| T1/2 | 38 | 25(65.8) | 13(34.2) |  |
| T3/4 | 82 | 50(60.9) | 32(39.1) |  |
| **LNM** |  |  |  | 0.497 |
| Yes | 74 | 48(64.9) | 26(35.1) |  |
| No | 46 | 27(58.7) | 19(41.3) |  |
| **Tumor size** |  |  |  | 0.333 |
| <6cm | 101 | 65(64.3) | 36(35.7) |  |
| >=6cm | 19 | 10(52.6) | 9(47.4) |  |
| **Differentiation** |  |  |  | **0.009** |
| Low/undifferentiated | 86 | 60(69.8) | 26(30.2) |  |
| High/moderate | 34 | 15(44.1) | 19(55.9) |  |
| **TNM stage** |  |  |  | 0.603 |
| I/II | 55 | 33(60) | 22(40) |  |
| III | 65 | 42(64.6) | 23(35.4) |  |
| Age*=mean age; LNM=lymph node metastasis | | | | |

The 120 patients were histologically diagnosed with gastric cancer. None of the included patients received any pre-surgery chemotherapy. Data were analyzed using Pearson’s chi-square test or Fisher’s exact test. All analyses were carried out by the use of SPSS18.0 software (SPSS Inc., Chicago, IL).
